# Contralateral nodal failures in oropharyngeal cancers after TORS and unilateral neck management: a retrospective study

**DOI:** 10.1186/s40463-021-00551-9

**Published:** 2021-12-23

**Authors:** Axel Sahovaler, John J. W. Lee, Wei Xu, Susie Su, Ali Hosni, Andrew Bayley, David P. Goldstein, John R. de Almeida

**Affiliations:** 1 Department of Otolaryngology Head and Neck Surgery, University of Toronto, 200 Elizabeth St., 8NU-883, Toronto, ON M5G 2C4 Canada; 2 Department of Biostatistics, University of Toronto, Toronto, ON Canada; 3 Department of Radiation Oncology, University of Toronto, Toronto, ON Canada; 4 Princess Margaret Cancer Center-University Health Network, Sinai Health System, Toronto, Canada

**Keywords:** TORS, Contralateral nodal failure, Oropharyngeal cancer

## Abstract

**Background:**

Report the incidence of contralateral nodal failure rates in well-lateralized oropharyngeal carcinoma treated with upfront surgery and unilateral neck management.

**Methods:**

Lateralized oropharyngeal carcinomas treated with upfront surgery using transoral robotic surgery (TORS) and unilateral neck management (unilateral neck dissection ± unilateral radiation treatment) were identified. Primary endpoint was contralateral regional control (CRC). Secondary endpoints were local control (LC), and overall survival (OS).

**Results:**

Thirty-two patients were included. Pathologic T categories included 66% pT1, 31% pT2 and 3% pT3. Nodal diseases comprised 41% N0 and 47% N1 (AJCC 8th). Twenty-three (72%) patients had HPV related tumors. 3-years CRC, LC and OS were 100%, 96% (89–100) and 96% (CI 89–100). One patient developed a second primary with contralateral nodal disease. Only one patient died from another primary cancer.

**Conclusion:**

In selected patients with lateralized oropharyngeal cancer, treatment with TORS and ipsilateral management of the neck may be oncologically safe without significant risk of contralateral failure.

*Level of evidence*: Level 2.

**Graphical abstract:**

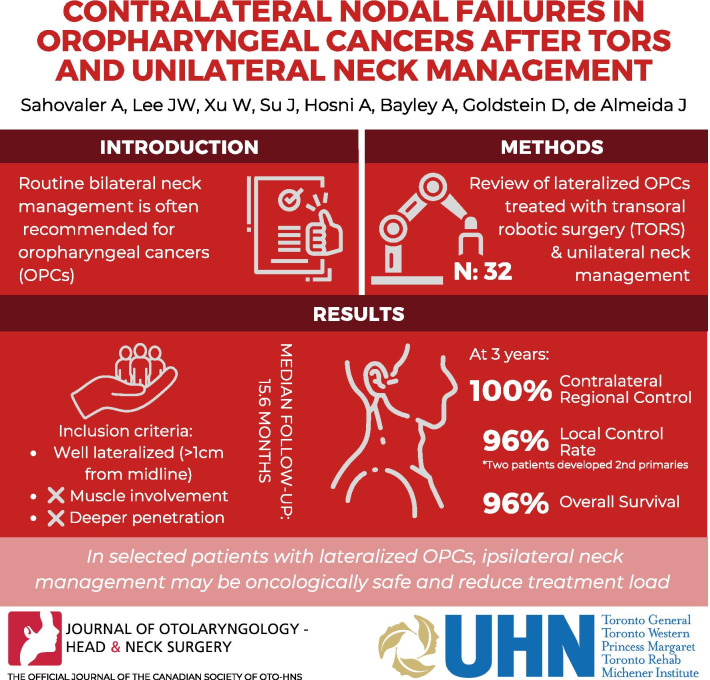

## Introduction

The presence of lymph node metastases in oropharyngeal squamous cell carcinoma (OPC) is relatively common, occurring in 84% of HPV positive and 66% of HPV negative mediated cancers [1]. The prevalence of contralateral or bilateral disease, however, is much more rare and can be observed in 14.2% of patients with HPV positive disease and 14.5% of patients with HPV negative disease [1]. Because of the rich lymphatics present in the oropharyngeal mucosa, the National Comprehensive Cancer Network (NCCN) [2] as well as the American Society for Radiation Oncology (ASTRO) [3] recommend routine bilateral management of the neck in patients with tongue base, soft palate, and pharyngeal wall tumors as well as tonsil tumours with tongue base extension. Due to these recommendations combined with a concern for regional failure in the contralateral untreated neck, many patients receive treatment despite no clinically apparent disease.

With the improved survival associated with human papilloma virus (HPV) mediated OPC [4–6], the head and neck community has begun to focus efforts on functional outcomes and reducing the toxicities of treatment. Two large recent trials examined the impact of substituting traditional cisplatin-based chemotherapy for cetuximab but failed to show an amelioration in toxicities with cetuximab and in fact demonstrated reduced survival [7, 8]. Other efforts have concentrated on reducing the dose of radiotherapy but few trials examine the role of reducing the volume of treatment.

Although many oropharyngeal cancers have historically required bilateral treatment, several studies have shown that unilateral management of the neck in highly selected patients treated with radiotherapy may be oncologically safe and feasible, particularly for patients with tonsil cancers isolated to the tonsillar fossa and with limited nodal disease [9–13]. Unilateral management of the neck is associated with potential reduction of treatment toxicity as the contralateral neck and parotid gland can be spared from the deleterious effects of the radiotherapy [14, 15]. As a result, reduced acute and late toxicities such as xerostomia and dysphagia and improved patient reported quality of life have been reported [16, 17]. This benefit in reduced treatment morbidity must be weighed against the risk of contralateral nodal recurrences [18]. Most experiences of unilateral neck treatment in OPC come from primary radiotherapy (RT) series, whereas data from primary surgical experiences about this topic is scarce. The objective of this study was to report the incidence of contralateral nodal failure rates in well lateralized OPCs after transoral robotic surgery (TORS) and unilateral neck treatment.

## Material and methods

Institutional research ethics board approval was obtained for the study. All tonsil and base of tongue squamous cell carcinomas treated with upfront surgery using TORS and unilateral neck management (with a neck dissection and/or radiation treatment) at the University Health Network in Toronto were identified from a prospectively maintained surgical database from November 2014 till January 2019. For our study we only included well lateralized OPCs, that is tumors located > 1 cm from the midline, without muscle involvement or any suspicion of deeper penetration. Regarding N category, tumors were N0 or N1 according to the AJCC 8th Edition [19]. Patients were also included if they underwent definitive resection of a lateralized oropharyngeal cancer during a diagnostic transoral robotic surgical approach for identifying unknown primary. For these patients, a neck dissection was not planned and the neck was staged based on imaging studies when neck dissection was not performed. Head and neck computed tomography (CT) and magnetic resonance imaging (MRI) were used for staging in every case.

### Surgery

For primary tumor resection all patients were approached using transoral robotic surgery (TORS) by two of the authors (JDA, DG). Patients with a known primary at the time of surgery underwent concomitant neck dissection with prophylactic branch ligation of the external carotid arteries (e.g. lingual artery and sometimes facial and ascending pharyngeal). In primary unknown tumors, the TORS approach was performed as part of the diagnostic algorithm to identify the primary. In these cases, neck disease was treated with unilateral definitive primary radiotherapy.

### Radiotherapy

When postoperative RT was utilised, only patients receiving unilateral treatment were analyzed. The decision to treat with adjuvant RT followed written institutional management policies and was discussed in a weekly peer-reviewed quality assurance round. Close or positive resection margins, perineural invasion, lymphovascular invasion, the presence of 2 or more involved lymph nodes, or a single node > 3 cm were indications for adjuvant treatment. A dose of 60 Gy was delivered in the adjuvant setting.

Immunohistochemical analysis for P16 status was performed on all specimens to identify HPV related tumors.

All patients underwent CT ± MR simulation. Radiation treatment commenced 6–8 weeks after post-operatively in all cases, using Intensity-Modulated Radiation Therapy (IMRT). Daily image guidance was performed to ensure precision of RT delivery.

### Follow-up

Routine surveillance was undertaken at 3-month intervals for the first 2 years; 4-month intervals in years 2 to 3, 6-month intervals for years 3 through 5, and annually there-after in a multidisciplinary setting according to institutional protocol. CT/MRI was performed 8 to 12 weeks after RT to assess treatment response. Local or regional failures were recorded based on histologic confirmation, and distant failure relied on radiologic and histologic evidence.

### Statistical analysis

Demographic information, comorbidities, substance abuse history, tumor characteristics (including p16 immunohistochemistry), surgical details and treatment modalities (adjuvant RT or CRT) were collected.

The primary endpoint of this study was contralateral neck regional control (CRC) defined as clinical evidence of metastatic cervical lymph nodes in the contralateral neck. Secondary endpoints were local control (LC), and overall survival (OS). LC was defined from date of diagnosis to date of local failure or last follow-up. Events were local failures. For OS, all deaths constituted events.

Frequency (percentage) were provided for categorical variables, while mean (SD) and median (min, max) were presented for continuous variables. Non-parametric Kruskal–Wallis tests were applied for comparisons of continuous variables while Chi Square tests were used for comparisons of categorical variables. Univariate survival analysis with the Kaplan–Meier method was used to calculate survival outcomes.

## Results

### Patient and tumor characteristics

Thirty-two patients with well-lateralized OPC and unilateral neck treatment were included for the final analysis. Median follow-up was 15.6 months (range 3.6–51.6). There were 23 (72%) patients with HPV related tumors, and 24 (75%) individuals had smoking history. Four patients (12%) had no clinical evidence of the primary tumor (cT0), and diagnosis was made after a TORS approach for a primary unknown cancer. Pathologic T categories included: 21 (66%) patients with pT1 disease, 10 (31%) with pT2 disease and one (3%) with pT3 disease. There were 13 (41%) patients classified as pN0 and 15 (47%) patients were classified as pN1. The four remaining ones were classified as pNx, which were the primary unknown tumors in which a neck dissection was not performed. Of note, 4 (13%) patients had multiple ipsilateral pathological nodes. Tumors were isolated to the tonsil in 14 (44%) patients and BOT in 7 (22%). In the remaining cases, tumors were located in the tonsil with BOT extension in 8 (25%) and tonsil with soft palate extension in 3 (9%). In patients with BOT tumors, invasion was limited to the mucosa with no deeper tumor extension. Neck dissections were carried out in 28 (88%) patients. Nine (28%) patients received radiotherapy, four of them (the ones who were initially approached as primary unknown cancers) for definitive management of nodal disease after primary tumor resection. In all cases, neck irradiation was ipsilateral to the primary. Chemoradiotherapy was utilised in one patient (3%) due to a positive margin during a robotic resection of an unknown primary tumor. Locations of primary unknown cancers were two in the tonsil and two in the BOT. Additional patient and tumor characteristics are summarized in Tables 1 and 2.

**Table 1:** Summary of the general characteristics of the cohort, classified by P16 status

Covariate	Full sample (n = 32) n (%)	P16 (-) (n = 9) n (%)	P16 ( +) (n = 23) n (%)	*p*-value
*Gender*				0.23
Female	9 (28)	4 (44)	5 (22)	
Male	23 (72)	5 (56)	18 (78)	
*Age*				0.69
Mean (sd)	58 (9.4)	56.5 (11.9)	58.6 (8.5)	
Median (Min,Max)	57.9 (37.1,74.1)	55.9 (37.1,74.1)	58 (44.2,71.5)	
*Smoking Hx*				0.092
Current	17 (53)	7 (78)	10 (43)	
Ex-smoker	7 (22)	2 (22)	5 (22)	
Non-smoker	8 (25)	0 (0)	8 (35)	
*Pack years*				**0.019**
Mean (sd)	22 (21.5)	36.2 (23.4)	16.4 (18.4)	
Median (Min,Max)	19 (0,80)	30 (10,80)	10 (0,60)	
*Drinking Hx*				< **0.001**
Ex-drinker	3 (9)	3 (33)	0 (0)	
Heavy	6 (19)	4 (44)	2 (9)	
Light	9 (28)	2 (22)	7 (30)	
Moderate	2 (6)	0 (0)	2 (9)	
Non-drinker	11 (34)	0 (0)	11 (48)	
Unknown	1 (3)	0 (0)	1 (4)	
*cT stage*				**0.035**
T0	4	0	4	
T1	19 (72)	9 (100)	10 (61)	
T2	9 (28)	0 (0)	9 (39)	
*cN stage*				**0.049**
N0	15 (47)	7 (78)	8 (35)	
N1	17 (53)	2 (22)	15 (65)	
*pT*				0.77
1	21 (66)	7 (78)	14 (61)	
2	10 (31)	2 (22)	8 (35)	
3	1 (3)	0 (0)	1 (4)	
*pN*				0.43
0	13 (41)	5 (56)	8 (35)	
1	15 (47)	4 (44)	11 (48)	
x	4 (12)	0 (0)	4 (17)	
*ENE*				0.32
No	27 (96)	8 (89)	19 (100)	
Yes	1 (4)	1 (11)	0 (0)	
Missing	**4**	**0**	**4**	
*PNI*				1
No	30 (94)	9 (100)	21 (91)	
Yes	2 (6)	0 (0)	2 (9)	
*LVI*				1
No	26 (84)	8 (89)	18 (82)	
Yes	5 (16)	1 (11)	4 (18)	
Missing	**1**	**0**	**1**	
*Histology grade*				**0.011**
Mod. diff	13 (68)	9 (100)	4 (40)	
Poorly diff	6 (32)	0 (0)	6 (60)	
Missing	**13**	**0**	**13**	
*Radiation treatment*				0.38
No	23 (72)	8 (89)	15 (65)	
Yes	9 (28)	1 (11)	8 (35)	
*Chemo*				1
No	31 (97)	9 (100)	22 (96)	
Yes	1 (3)	0 (0)	1 (4)	
*Site*				0.65
BOT	6 (19)	1 (11)	5 (22)	
Tonsil	26 (81)	8 (89)	18 (78)	
*Follow-up (months)*				0.98
Mean (sd)	21.6 (15.6)	21.6 (14.4)	21.6 (15.6)	
Median (Min, Max)	15.6 (3.6, 51.6)	26.4 (3.6, 46.8)	13.2 (4.8, 51.6)	

Statistically significant values are given in bold

**Table 2:** Subsite/nodal classification of the cohort

	N0	N1 (%)	Total (%)
Isolated tonsillar fossa	7 (22)	7 (22)	14 (44)
Isolated BOT	3 (9)	4 (12)	7 (22)
Tonsil + BOT	2 (6)	6 (19)	8 (25)
Tonsil + Soft palate	3 (9)	–	3 (9)
Total	15 (47)	17 (53)	32 (100)

*BOT* base of tongue

### Contralateral neck regional control

There were no isolated contralateral neck failures in the cohort, and CRC for those followed out to 3-years was 100%. A patient which presented a T2N0 p16 positive left tonsil primary tumor treated with TORS and neck dissection with clear margins and no adjuvant therapy, developed a second primary in the midline tongue base with nodal disease contralateral to the original disease 4 years after the original surgery. We categorized this as a secondary primary tumor therefore we did not include this as a contralateral neck failure.

### Local control and overall survival

**Fig. 1:**
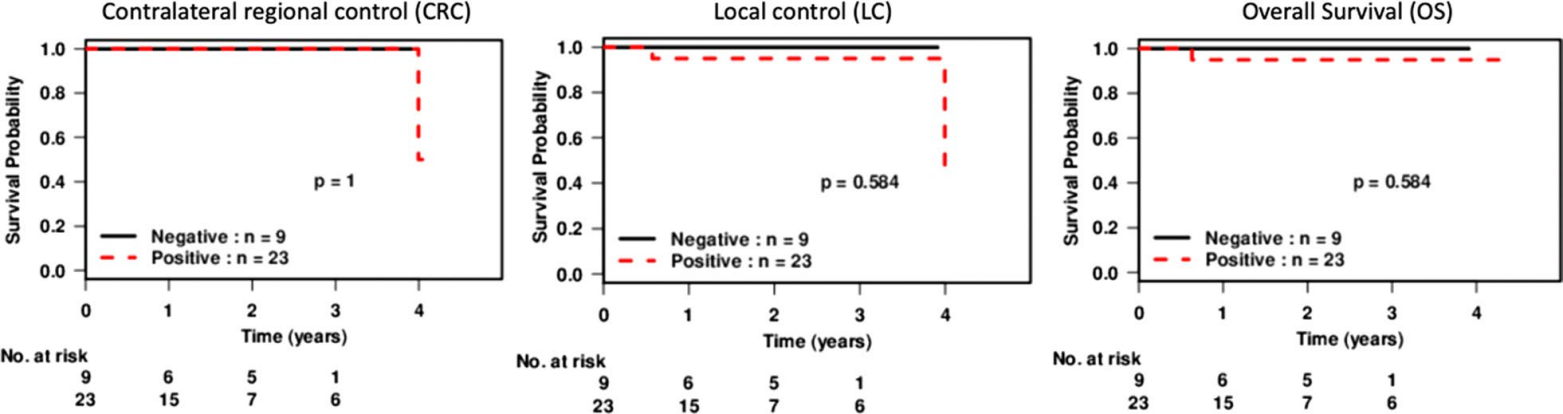
Kaplan–Meier curves demonstrating contralateral regional control (CRC), local control (LC) and overall survival (OS)

Three-year LC rates were 96% (89–100). Two patients (6%) developed second primary tumors, one mentioned above and a second with p16 negative T1N0 tonsil cancer who developed a tumor in the uvula and soft palate which was treated with radiotherapy (Fig. 1). In both cases, tumors were disparate from the original site. OS at 3 years was 96% (CI 89–100), for HPV negative 100% (CI 100–100) and for HPV positive 95% (CI 86–100). One patient died from another primary cancer (rectal) 7 months after completion of treatment, but had no evidence of primary site disease.

## Discussion

We present the results of a single institutional cohort of patients treated primarily through a surgical approach with ipsilateral management of the neck in well-lateralized oropharyngeal cancer. We observed no isolated contralateral neck recurrences in our cohort, which included patients with tumors of the tongue base and tumors of the tonsil with tongue base and soft palate invasion. Over half of the present cohort would typically receive bilateral neck treatment based on ASTRO treatment recommendations. This data suggests that in carefully selected patients this approach may be oncologically safe and patients may benefit from avoidance of treatment of the contralateral neck with potential for a reduction in treatment related toxicities. Unfortunately, we did not have prospectively collected toxicity or functional outcome data. Our study contributes to the literature in a contemporary discussion about elective management of the contralateral neck in OPC, and reports the outcomes of a primary surgical approach with a minimally invasive technique.

Our group was one of the pioneering institutions for management of tonsil cancers with unilateral neck radiotherapy [20], most recently reporting that only 2% of patients experienced a contralateral regional failure in well-lateralized tonsillar cancer [21], with higher rates of gastrostomy tube dependency at 12 months with bilateral RT compared to the unilateral approach (4.3% [2.3–7.2] vs 0%). The oncologic safety combined with improvements in side effects from treatment constitutes the cornerstone of this recent concept and has been supported by other reports. In a critical review of the literature, Al-Mamgani et al. [22] observed a mean incidence of contralateral failures of 2.42% after pooling data from 1116 patients with tonsil cancers treated with a unilateral radiotherapy approach, and discovered that involvement of the midline showed the most significant correlation with the incidence of contralateral nodal recurrence (12% with midline involvement vs 1.71% when midline was free *p* = 0.001). The authors also demonstrated, however, that patients who develop isolated contralateral recurrences can be successfully salvaged with neck dissection and/or radiation in 79% of cases. Noteworthy, and as it was observed in our cohort, there is a possibility that contralateral recurrences are due to a new primary which can sometimes be occult. Current practice guidelines recommend management of the contralateral neck in patients with multiple ipsilateral nodes and that unilateral approaches should be reserved for T1-T2 tonsillar cancers isolated to the tonsillar fossa or with minimal soft palate extension but without clinical or radiographic evidence of extracapsular extension [3]. A careful discussion should weigh the preferences of the patient and the relative benefits of unilateral treatment versus the potential for contralateral nodal recurrence and subsequent salvage treatment [3].

Most available evidence about this topic comes from patients treated with definitive (chemo)radiotherapy approach. There is a paucity of reports about unilateral approaches after primary surgery. Al-Mamgani et al. [22] suggested that data from surgical series might not be directly translated to consensus recommendations because of the theoretical concerns of altered lymphatic drainage patterns and contamination from surgical removal and manipulation of tissues, which may increase the risk of contralateral neck recurrence. Nevertheless, there is slowly growing recent data refuting this theory. Rackley et al. [23] presented a large cohort of 81 patients with squamous cell carcinomas of the tonsil treated with primary surgery and adjuvant RT delivered to the ipsilateral neck. The authors reported no contralateral recurrences. From a total cohort of 154 patients, Chin et al. [16] analyzed locoregional recurrence rates in 48 patients with lateralized tonsillar cancers, with N0 to N2b disease treated with surgery and ipsilateral RT. There were no neck recurrences, either ipsilateral or contralateral, in this study. Of note, approximately 51% of the total patients had transoral laser microsurgery (TLM) and concurrent chemotherapy was administered in 54% of the cases treated ipsilaterally in contrast to our study, with only one patient requiring chemotherapy. These data would suggest that the relatively low risk of contralateral neck failure is not accounted for by the addition of chemotherapy in the adjuvant setting. In addition, after including patients with BOT tumors, our results elicit that ipsilateral management may be expanded to include tumors with limited extension of the tongue base or highly selected lateralized tongue base cancers.

Perhaps the greatest argument in favour of a unilateral approach is to mitigate the toxicities of therapy. For patients managed surgically, neck dissection is often associated with impairment of shoulder function and bilateral neck dissection similarly has increased morbidity to the contralateral shoulder [24–26]. Often patients treated surgically require adjuvant therapy and even if they undergo an ipsilateral neck dissection, adjuvant therapy is delivered to the contralateral nodal basin to minimize risk of contralateral failures. The reduction in both acute and late toxicities with ipsilateral treatment have been well described in the literature. Chin et al. [16] described significantly lower acute toxicity scores, particularly in mucositis, xerostomia and weight loss, as well better patient reported outcomes in the MD Anderson Dysphagia Inventory (MDADI) [27] and the University of Michigan Xerostomia Questionnaire (XQ) [28] in patients irradiated unilaterally, in comparison to the ones who received bilateral RT. In a matched pair analysis, Kim et al. also demonstrated differences in the occurrence of grade ≥ 2 acute and late toxicities favoring the unilateral approach vs bilateral radiation treatment (45.7% vs. 74.3% *p* = 0.001 and 7.1% vs 18.6% *p* < 0.001).

The findings from the present study suggest that a site and proximity to midline -based strategy may be used in future trials to determine whether it may be safe avoid treatment to the contralateral neck. A number of studies have suggested that number of involved nodes [29, 30], and proximity to midline are the most important factors in deciding on the risk of contralateral failure [22, 31, 32]. A recent cooperative group prospective trial [33], demonstrated a relatively high negative predictive value (0.87) in patients with T2-T4 tumors in predicting the absence of disease in the N0 neck using PET scans. However, one caveat of this study is a false negative rate or occult disease rate of 13%. While PET alone may not be adequate to decide on who can avoid neck treatment, the addition of lymphatic mapping with peri-tumoral injections of the primary tumor followed by acquisition of SPECT-CT or planar lymphoscintigraphy may improve our ability to define lymphatic drainage patterns of tumors. A study out of the Netherlands Cancer Institute recently completed the accrual of a prospective trial where on lateralized head and neck cancers, SPECT-CT was employed to determine contralateral drainage [34]. In this study, patients without contralateral neck drainage received unilateral therapy and those with contralateral drainage received treatment only to the nodal levels that had uptake of the radiotracer. The final analysis of this study is pending, and opens a whole new landscape of future research where will be possible to tailor treatment depending on the risk of contralateral spread of the disease.

We acknowledge several limitations of this study. Firstly, the retrospective nature of the study makes description of toxicities related to treatment difficult and for this reason we have chosen not to report them. Secondly, post-hoc delineation and mapping of the actual extent of tumor involvement is difficult in order establish a precise definition of what constitutes a lateralized cancer. Furthermore, the small sample size precluded statistical comparisons for example between patients with tonsil and BOT cancers or other risk factors such as extracapsular nodal extension or perineural/lymphovascular invasion. Finally, there was no comparison group with patients treated with traditional bilateral RT approaches.

## Conclusion

In selected patients with lateralized oropharyngeal cancer, treatment with TORS and ipsilateral management of the neck may be oncologically safe without significant risk of contralateral failure. Future trials may investigate expansion of indications for unilateral neck management and more novel techniques to predict the risk of nodal metastasis to the contralateral neck.

## Data Availability

Anonymized data is available upon request.
